# Supplementary material for: Integration of spatial and single-nucleus transcriptomics to map gene expression in the developing mouse kidney
Source: Development. 2025 Dec 22;152(24):dev205003. doi: 10.1242/dev.205003 (PMC12772959; doi:10.1242/dev.205003)
Supplement: Supplementary information [file develop-152-205003-s1.pdf]

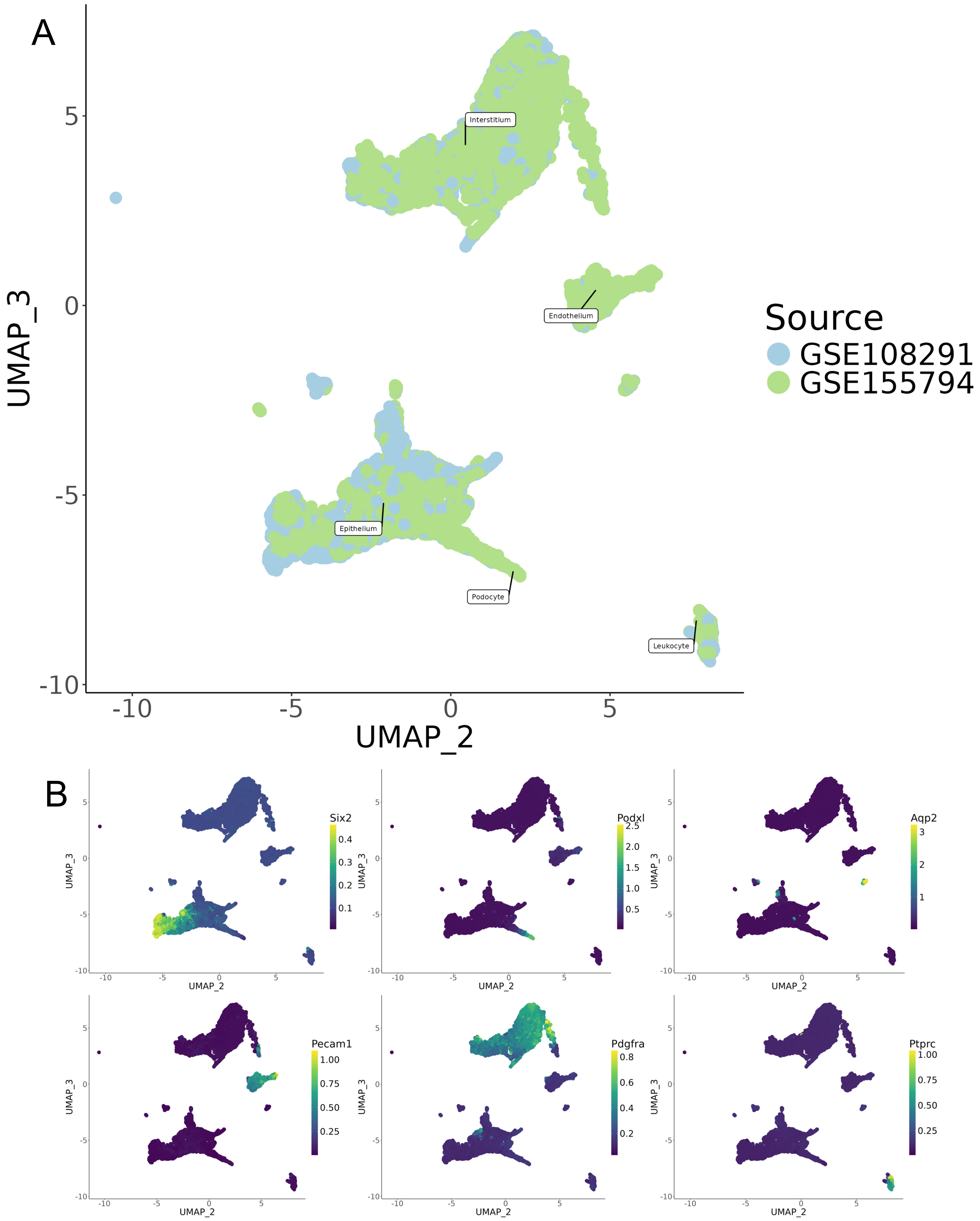

Development • Supplementary information

Fig. S1. Identification of Landmark Cell Types using Single-Cell Transcriptomics
(A) Successful integration of publicly available embryonic day 18.5 mouse kidney single-cell RNA-seq datasets (GEO accession numbers GSE108291 and GSE157594) is shown in the combined UMAP plot. (B) Coarse annotation of the integrated dataset based on the expression of canonical marker genes, which are expected to be cell-type specific.

Development • Supplementary information

Figure S2A

E15.5

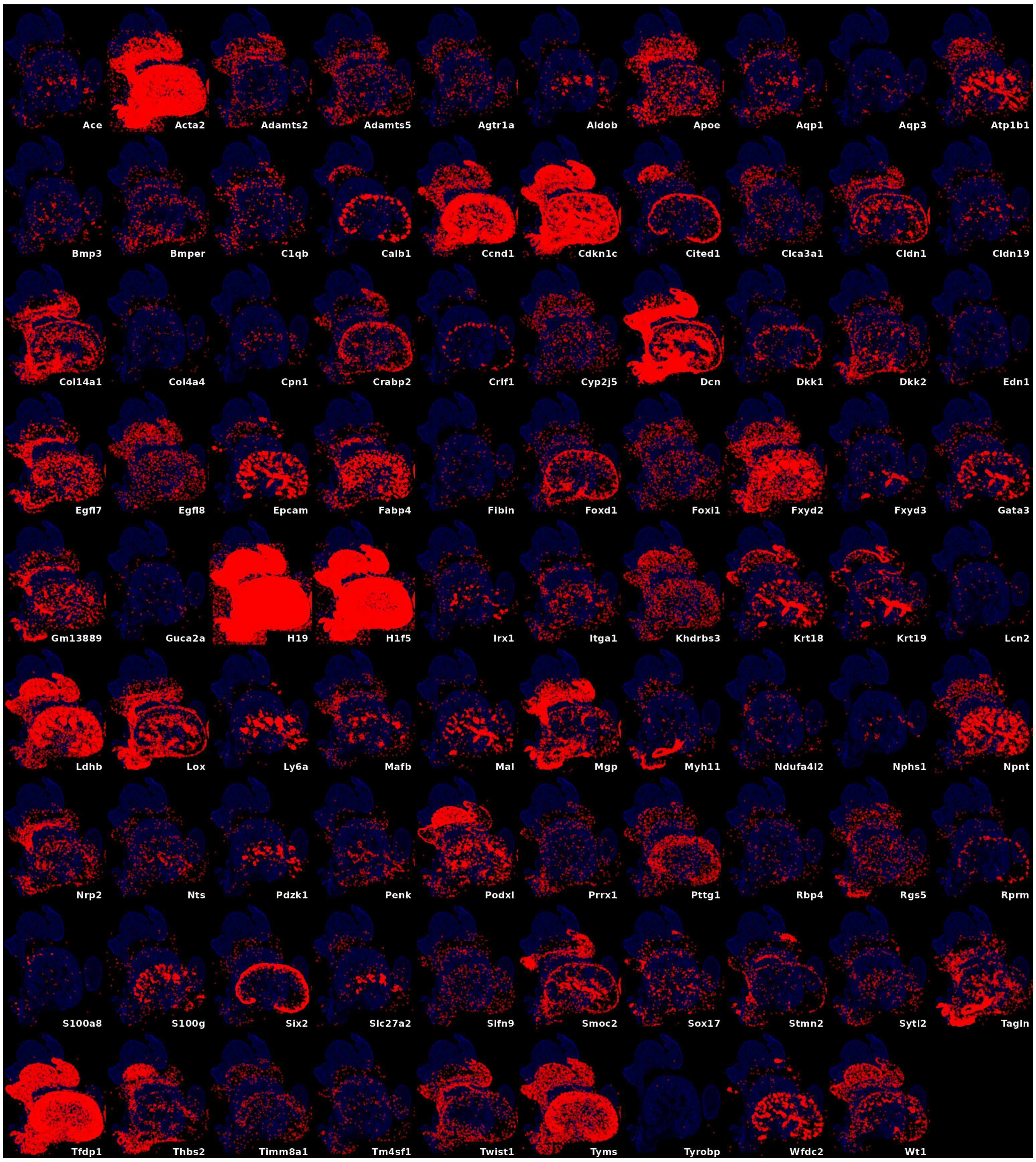

Development • Supplementary information

Figure S2B

E18.5

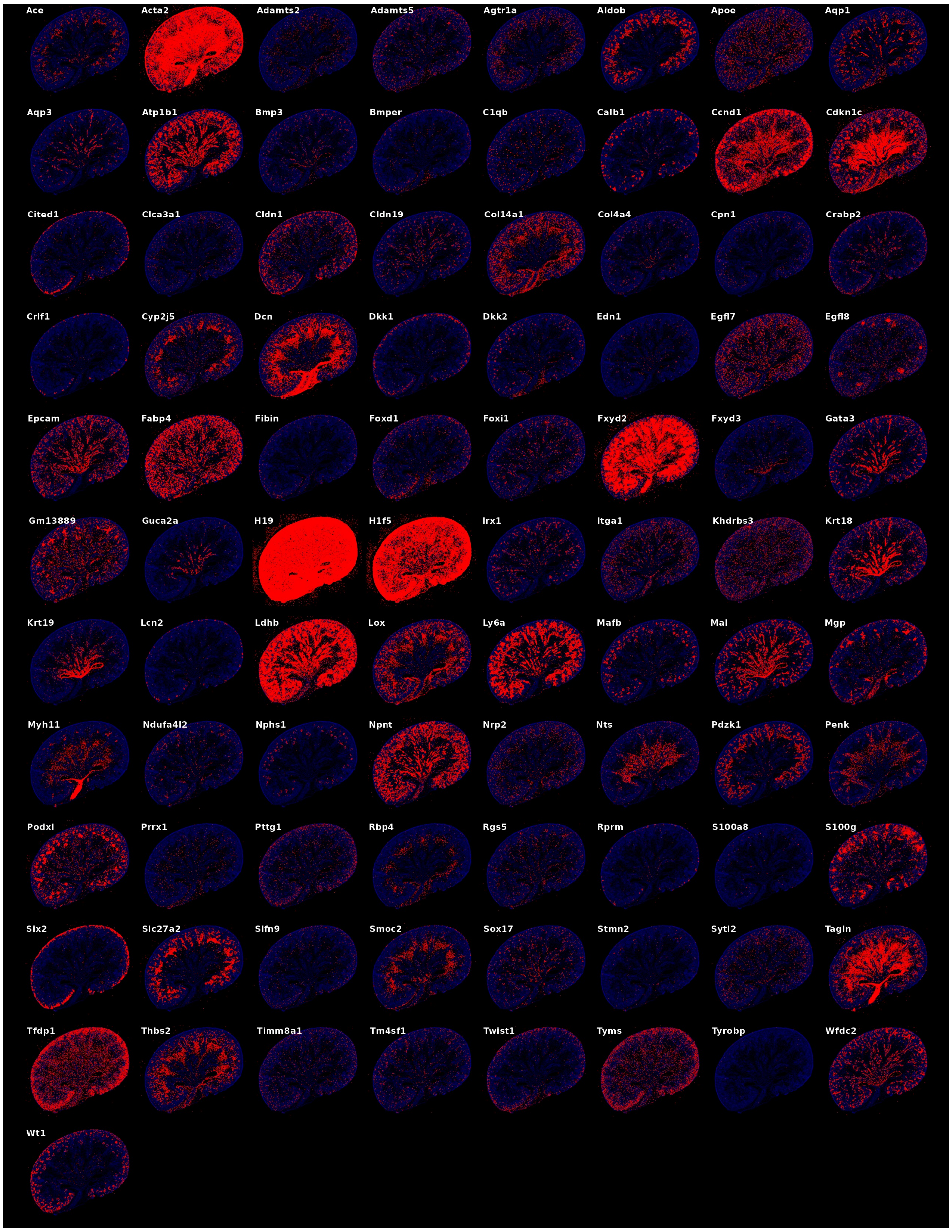

Development • Supplementary information

Figure S2C

P3

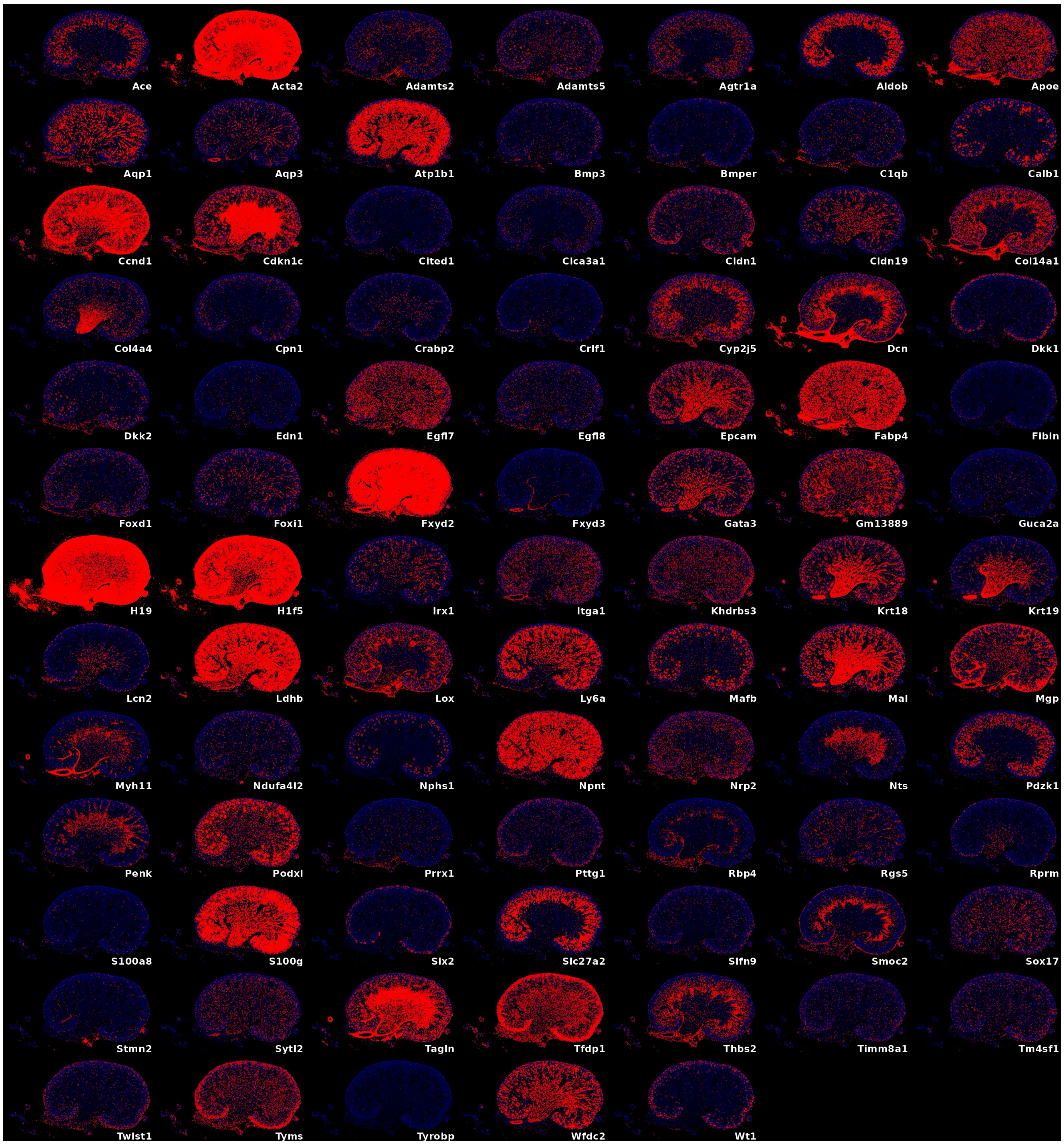

Fig. S2. Landmarks measured by CARTANA.

Spatial localization of landmark genes measured by CARTANA (A, E15; B E18, C P3). The figure shows in-situ sequencing signal of all landmark genes, grouped according to their targeted cell type. Red markers indicate the spatial location of landmark genes on a DAPI-stained background image, using coordinates from CARTANA results.

Development • Supplementary information

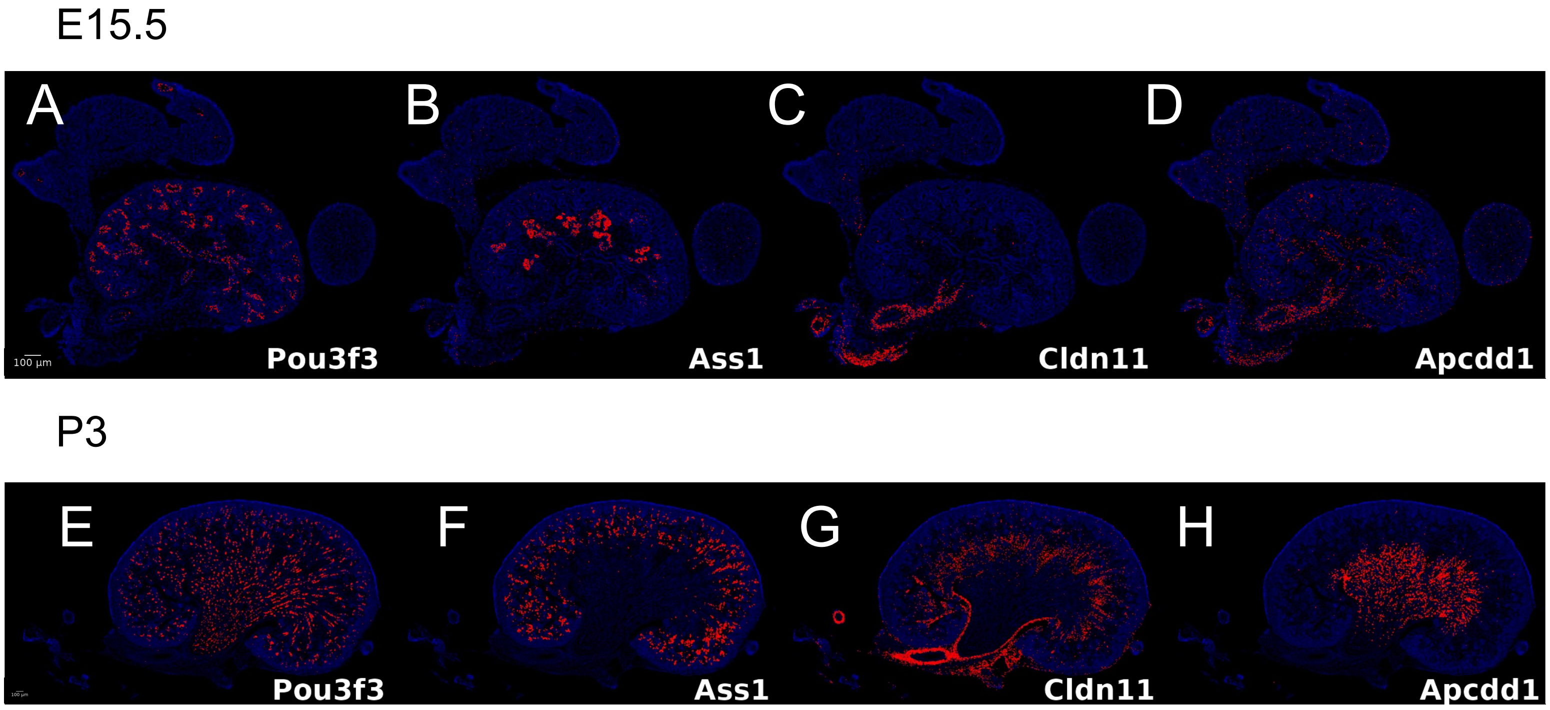

Fig. S3. Prediction of spatial expression at E15 and P3.

The predicted spatial distributions of Pou3f3, Ass1, Cldn11 and Apcdd1 (depicted at E18.5 in Figure 3) at the E15 (A-D) and P3 (E-H) stages indicating the extensibility of KSTAT.

Development • Supplementary information

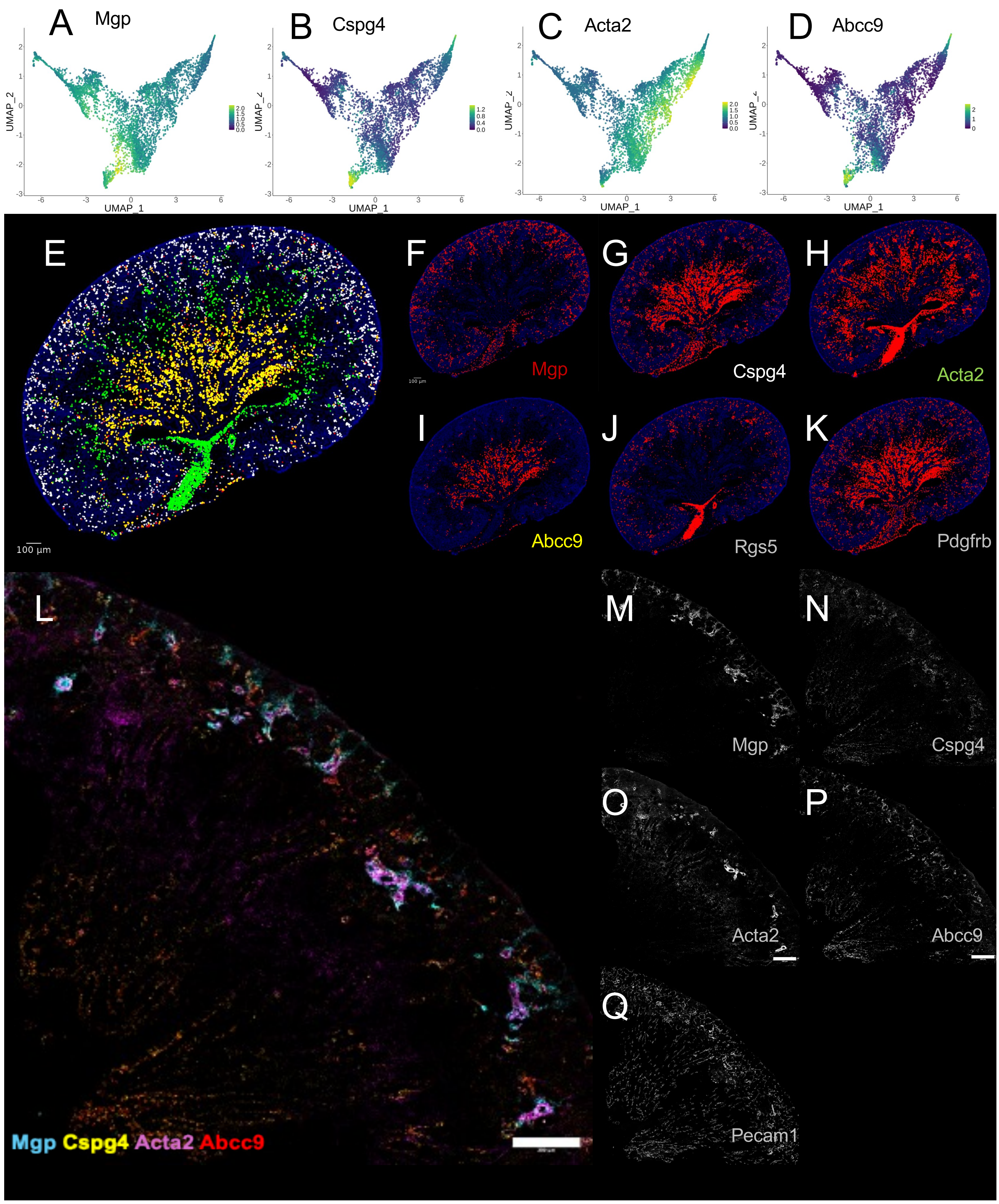
A Mgp
B Cspg4
C Acta2
D Abcc9
UMAP_1
UMAP_2
E
F
G
H
I
J
K
Mgp
Cspg4
Acta2
Abcc9
Rgs5
Pdgfrb
100 µm
L
M
N
O
P
Q
Mgp Cspg4 Acta2 Abcc9
Mgp
Cspg4
Acta2
Abcc9
Pecam1

Fig. S4. Heterogeneous spatial expression of mural cell marker genes.
(A-D) EIGEN analysis predicted that the genes Mgp, Cspg4, Acta2 and Abcc9 would demonstrate relative specificity for the four clusters of putative mural cells identified in Figure 7. Here we present UMAP's of isolated interstitial cells colored by expression for these genes.
(E) The predicted spatial expression of these four genes is simultaneously projected onto the 2D E18.5 kidney section (Mgp, red; Cspg4, white; Acta2, orange; Abcc9, yellow).
(F-I) The individual predicted spatial expression for the four genes.
(J-K) The predicted spatial expression for the canonical mural cell markers Rgs5 and Pdgfrb are shown for comparison.
(L) A composite image of RNAscope measurement of mRNA expression for Mgp, Cspg4, Acta2 and Abcc9 in E18.5 kidney that is largely consistent with the predictions by KSTAT, but implies additional heterogeneity.
(M-P) Greyscale images of the individual RNAscope measurement channels for Mgp, Cspg4, Acta2 and Abcc9.
(Q) Greyscale image of the mRNA expression of endothelial marker Pecam1 measured with RNA scope to demonstrate the location of blood vessels in the E18.5 kidney.

Development • Supplementary information

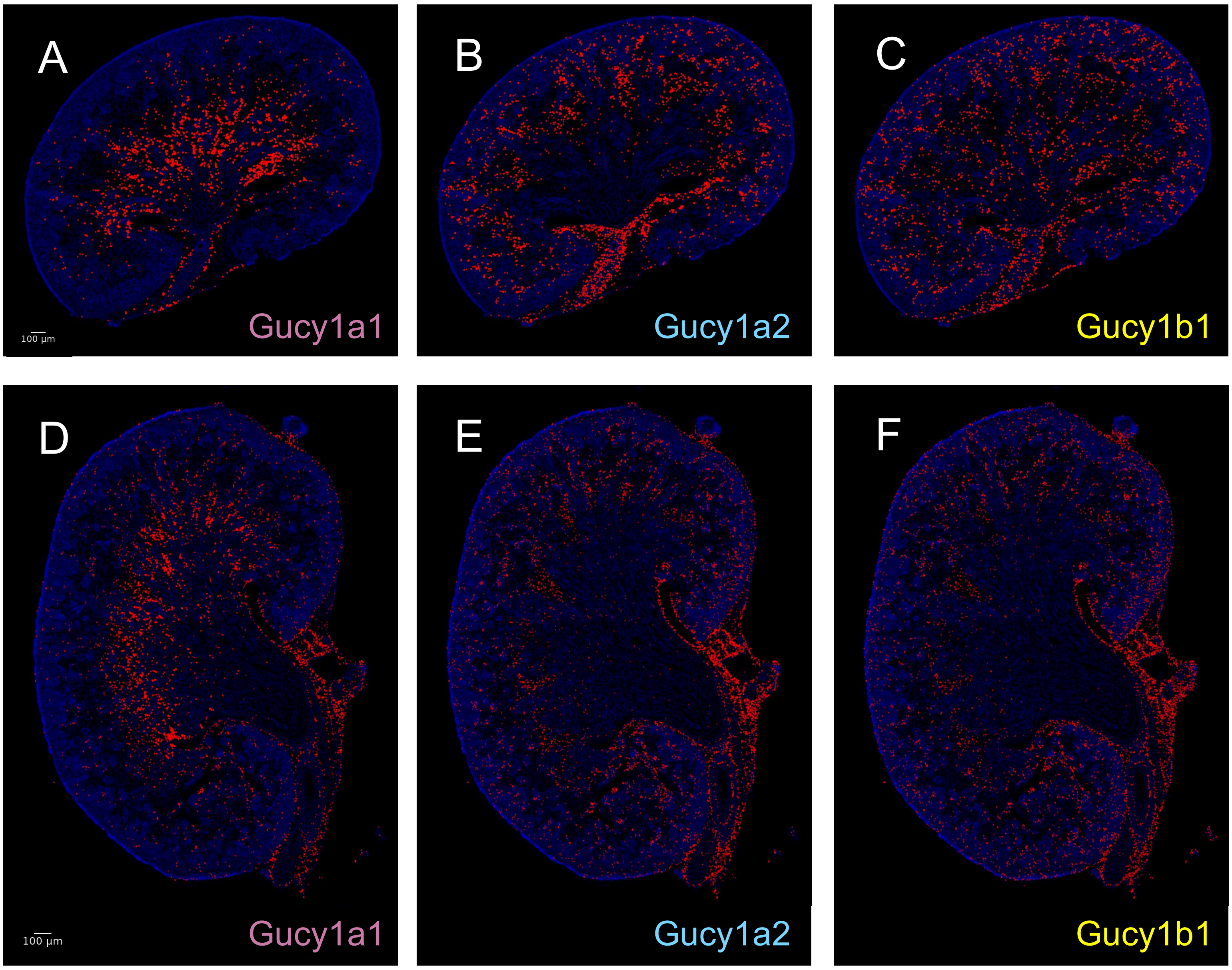

Fig. S5. Spatial heterogeneity of expression of soluble guanyl cyclases.
(A-E) Predicted spatial expression of the individual components of the heterodimeric soluble guanyl cyclases at E18.5 (A-C) and P3 (D-F). The coloring of the gene symbols corresponds to that of the points indicating predicted expression of the gene in Fig. 5.

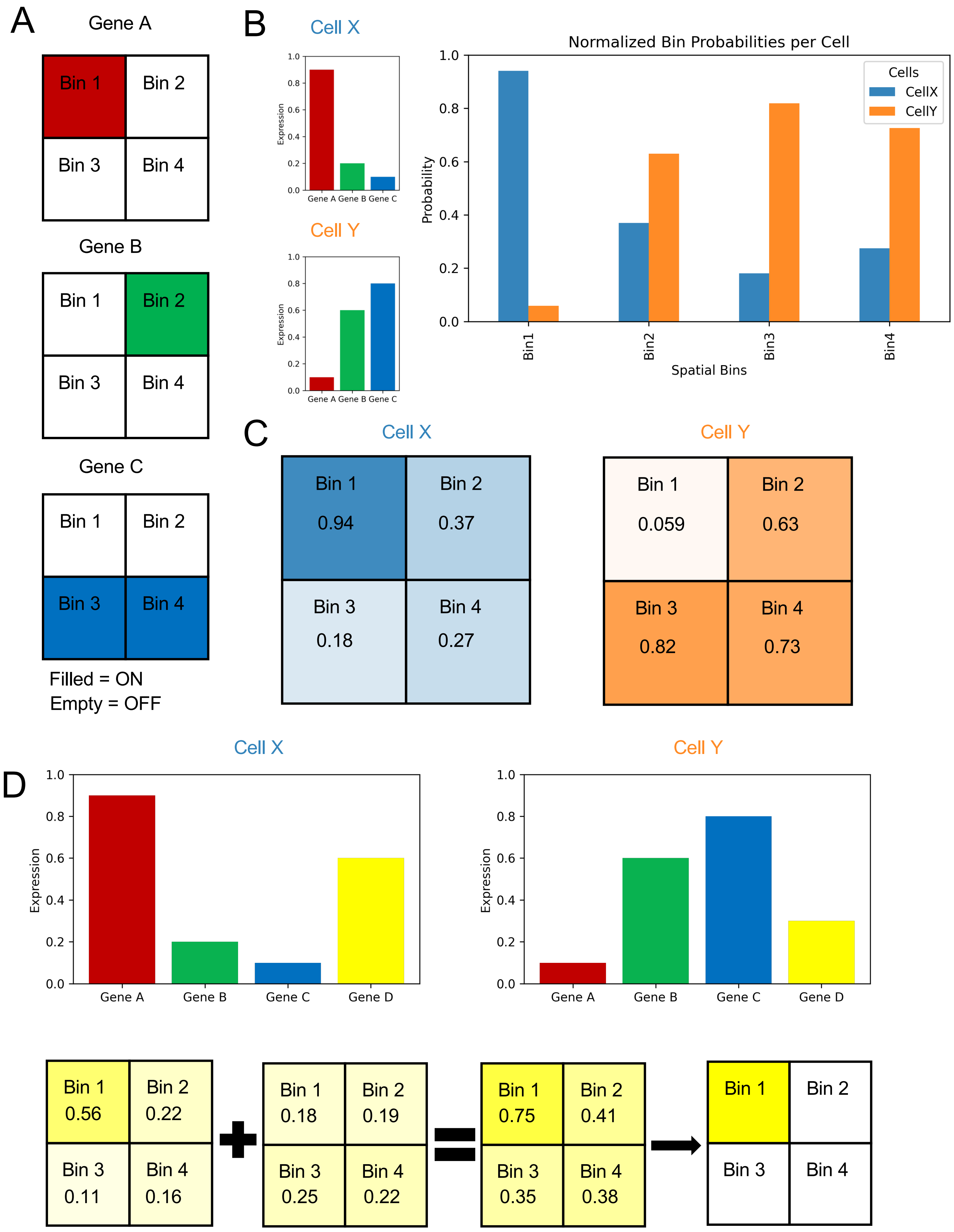

Development • Supplementary information

Fig. S6. Inferring the spatial origin of single nuclei using landmark gene expression.
We use landmark genes—genes with known spatial expression patterns—to estimate where each single nucleus might have originated within a reference tissue section. By comparing the expression of these genes in individual nuclei (measured by snRNA-seq) to the spatial patterns observed in a reference map, we can compute the probability that each nucleus came from different regions of the tissue. This allows us to visualize where cell types and molecular activities are likely located in the tissue, even though the cells were sequenced outside of their spatial context.
(A) The reference tissue is divided into spatial regions ("bins"), each defined by whether specific landmark genes (A–C) are active (ON) or inactive (OFF).
(B) Each nucleus from the snRNA-seq data is profiled for the same landmark genes. The expression pattern of each nucleus is compared to all spatial bins to find which locations it most closely matches.
(C) These match scores are converted into probabilities, creating a spatial probability distribution for each nucleus—that is, an estimate of how likely the nucleus came from each location in the tissue.
(D) Once spatial probabilities are assigned, other molecular features of each nucleus—such as the expression of non-landmark genes (here Gene D), transcription factor activity, or pathway enrichment—can be projected onto the tissue map by averaging across the cells in each bin. A threshold can then be applied to highlight the most relevant or confident regions.

Development • Supplementary information

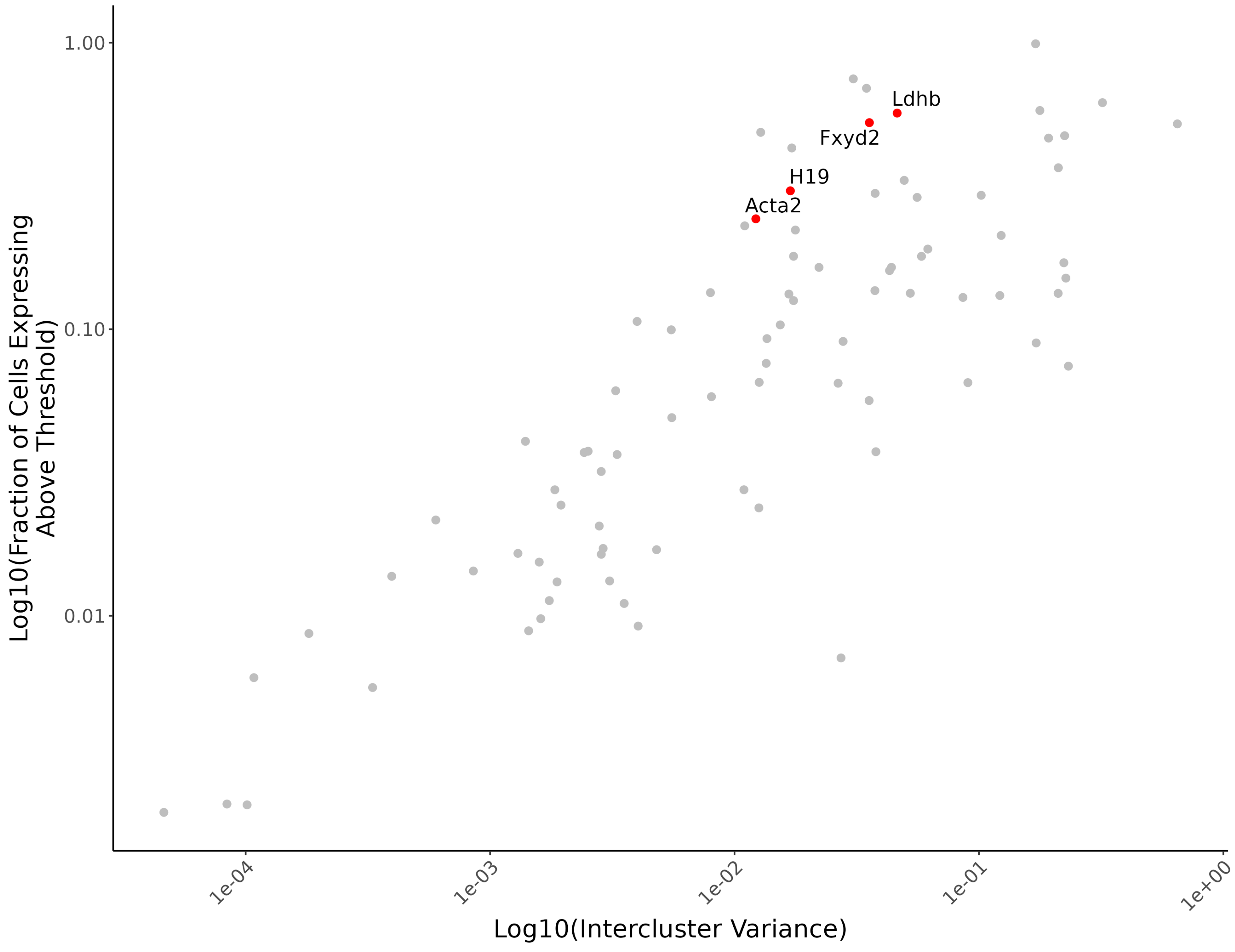

Fig. S7. Relationship between proportion of cells expressing landmark and intercluster variability. The four landmark genes with the highest total measured signal in the in situ sequencing experiment are characterized by a higher proportion of nuclei expressing the gene above a threshold relative to the intercluster variance of the gene's expression in the snRNA-seq dataset.

Development • Supplementary information

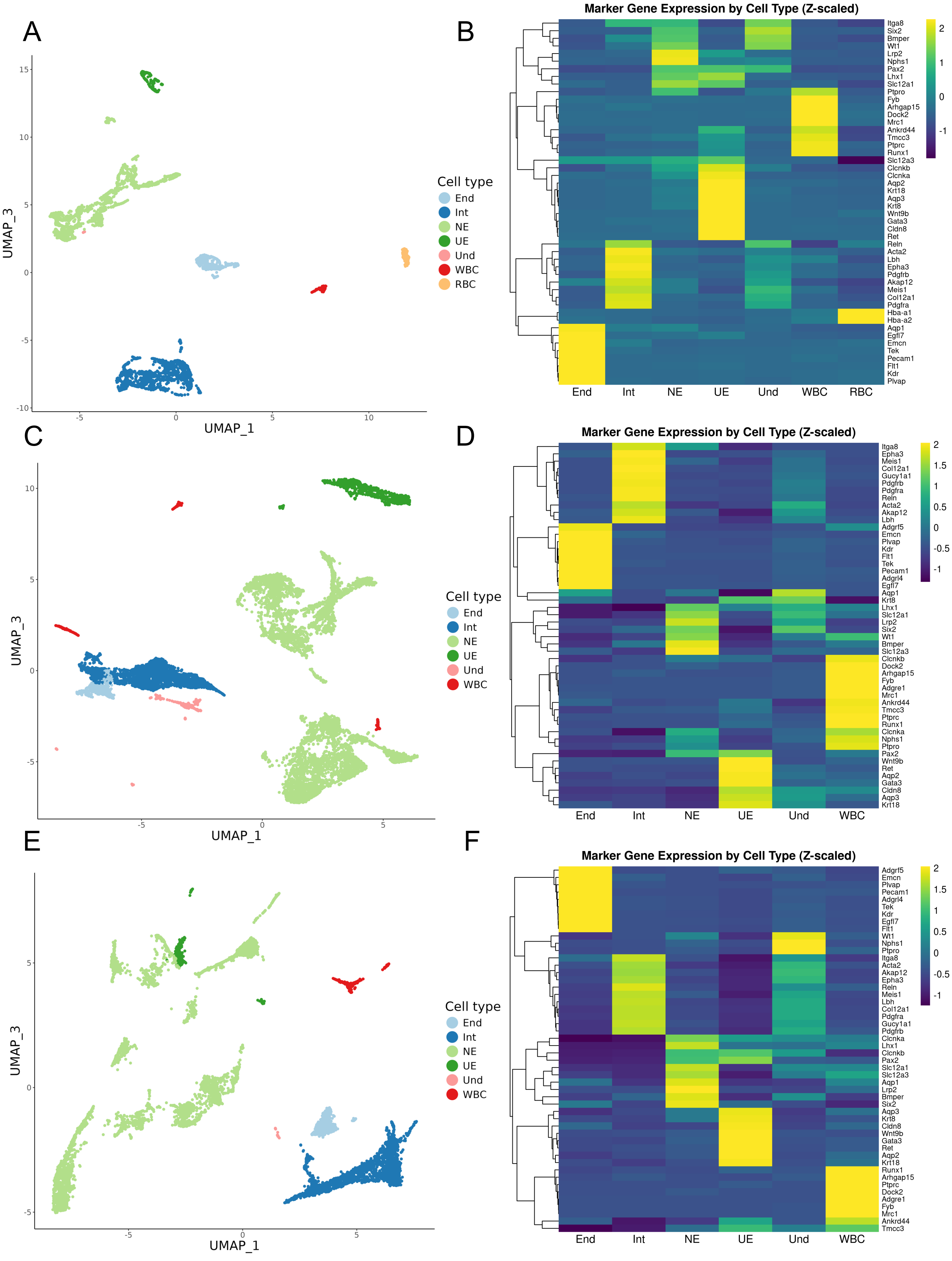
A
UMAP_3
UMAP_1
Cell type
End
Int
NE
UE
Und
WBC
RBC
B
Marker Gene Expression by Cell Type (Z-scaled)
Itga8 Six2 Bmper Wt1 Lrp2 Nphs1 Pax2 Lhx1 Slc12a1 Ptpro Fyb Arhgap15 Dock2 Mrc1 Ankrd44 Tmcc3 Ptprc Runx1 Slc12a3 Clcnkb Clcnka Aqp2 Krt18 Aqp3 Krt8 Wnt9b Gata3 Cldn8 Ret Reln Acta2 Lbh Epha3 Pdgfrb Akap12 Meis1 Col12a1 Pdgfra Hba-a1 Hba-a2 Aqp1 Egfl7 Emcn Tek Pecam1 Flt1 Kdr Plvap
End Int NE UE Und WBC RBC
C
UMAP_3
UMAP_1
Cell type
End
Int
NE
UE
Und
WBC
D
Marker Gene Expression by Cell Type (Z-scaled)
Itga8 Epha3 Meis1 Col12a1 Gucy1a1 Pdgfrb Pdgfra Reln Acta2 Akap12 Lbh Adgrf5 Emcn Plvap Kdr Flt1 Tek Pecam1 Adgrl4 Egfl7 Aqp1 Krt8 Lhx1 Slc12a1 Lrp2 Six2 Wt1 Bmper Slc12a3 Clcnkb Dock2 Arhgap15 Fyb Adgre1 Mrc1 Ankrd44 Tmcc3 Ptprc Runx1 Clcnka Nphs1 Ptpro Pax2 Wnt9b Ret Aqp2 Gata3 Cldn8 Aqp3 Krt18
End Int NE UE Und WBC
E
UMAP_3
UMAP_1
Cell type
End
Int
NE
UE
Und
WBC
F
Marker Gene Expression by Cell Type (Z-scaled)
Adgrf5 Emcn Plvap Pecam1 Adgrl4 Tek Kdr Egfl7 Flt1 Wt1 Nphs1 Ptpro Itga8 Acta2 Akap12 Epha3 Reln Meis1 Lbh Col12a1 Pdgfra Gucy1a1 Pdgfrb Clcnka Lhx1 Clcnkb Pax2 Slc12a1 Slc12a3 Aqp1 Lrp2 Bmper Six2 Aqp3 Krt8 Cldn8 Wnt9b Gata3 Ret Aqp2 Krt18 Runx1 Arhgap15 Ptprc Dock2 Adgre1 Fyb Mrc1 Ankrd44 Tmcc3
End Int NE UE Und WBC

Fig. S8. Transcriptomic datasets used for spatial inference.
(A, C, E) Combined single-cell RNA-seq data from E15.5 mouse kidney (A) and single-nucleus RNA-seq data from E18.5 (C) and P3 (E) were used as input to KSTAT to infer spatial gene expression in the developing kidney. UMAPs illustrate transcriptomic diversity, with cells colored by annotated cell type. All expected kidney populations are represented, including endothelium (End), interstitium (Int), leukocytes (WBC), nephron epithelium (NE), ureteric epithelium (UE), undetermined cells (Und), and erythrocytes (RBC).
(B, D, F) Heatmaps showing the mean expression of representative marker genes for each cell type at the corresponding developmental stage.

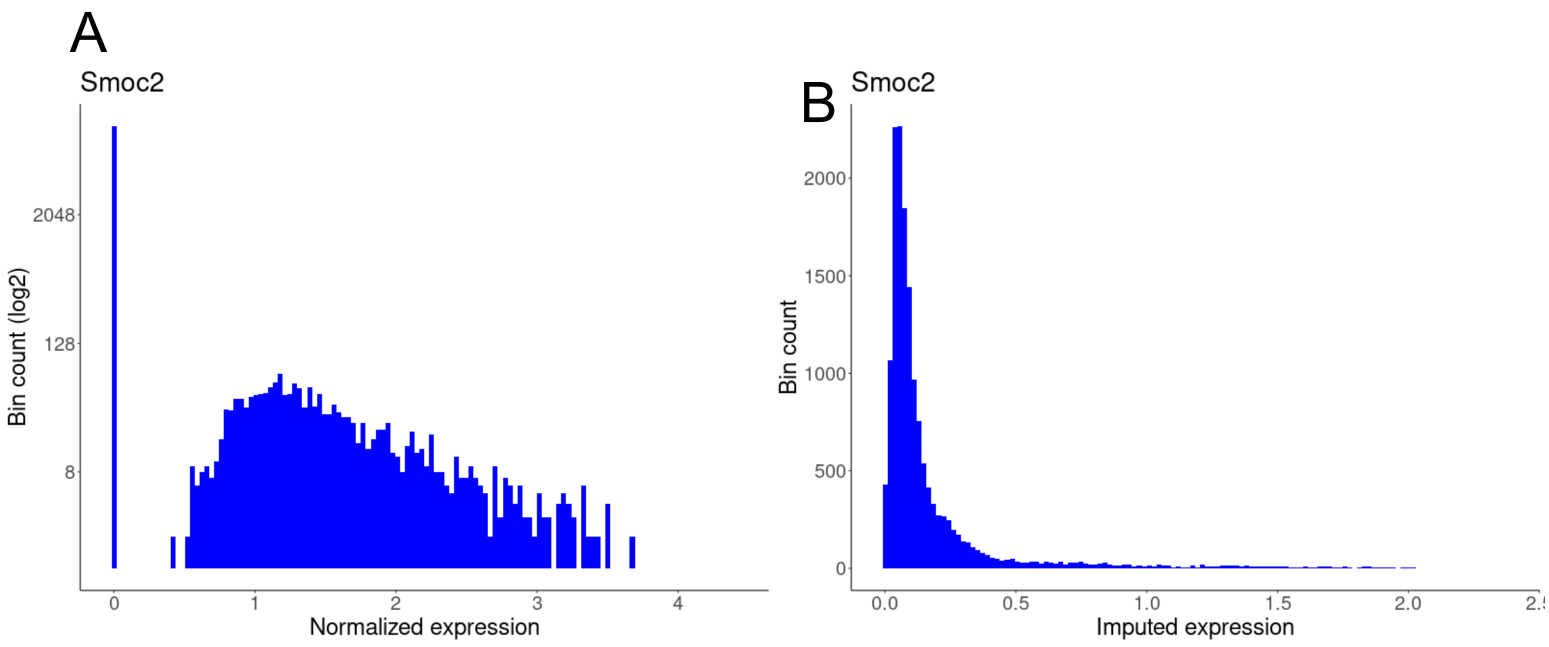

Fig. S9. Distributions of normalized and imputed expression values.

In panel A we show the distribution of normalized counts for the landmark Smoc2. Note that the y-axis is on a log-scale. This was necessary for visual clarity since the data is zero-inflated. In panel B we show distribution of expression values imputed with MAGIC. This data can be more reasonably modeled as a mixture of two Gaussian distributions.

Development • Supplementary information

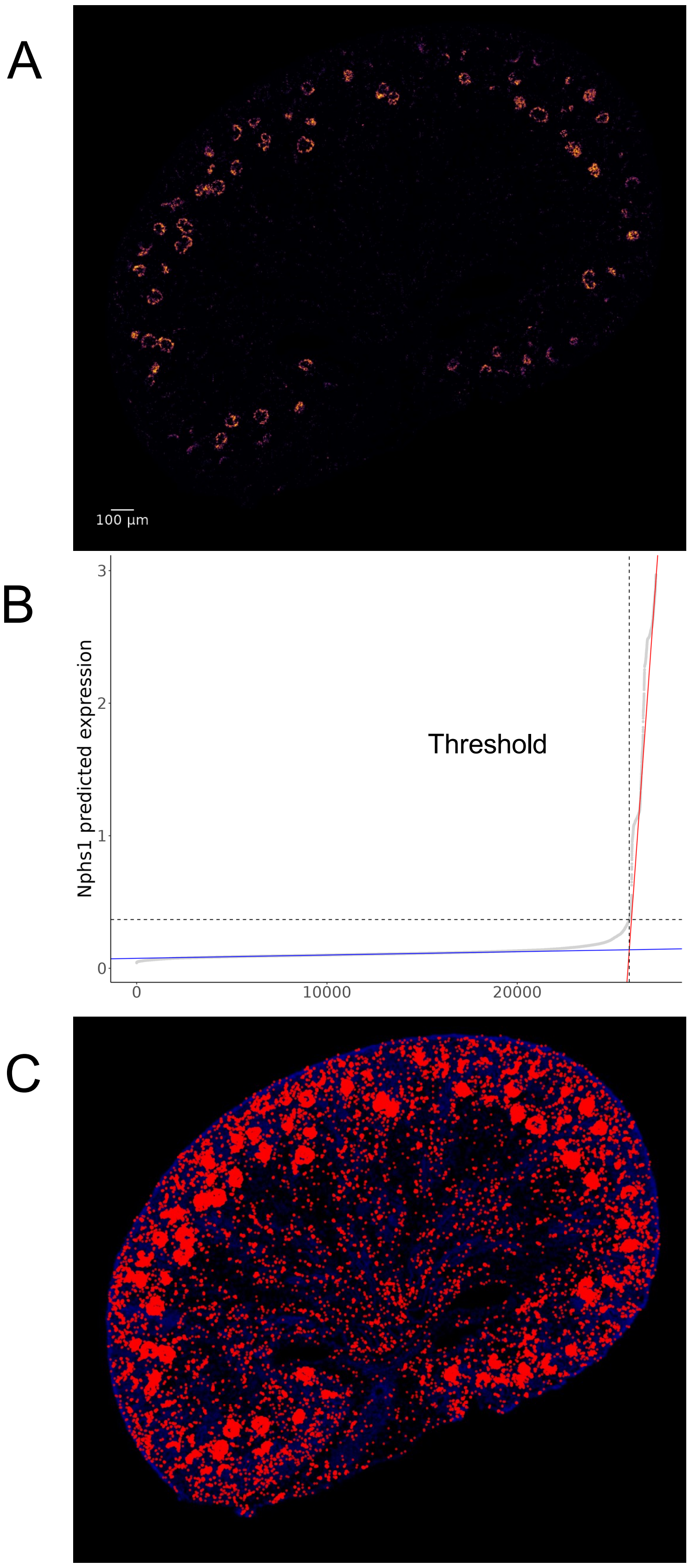

Fig. S10. Automatic Thresholding of Expected Gene Expression

(A) Expected expression of Nphs1 calculated using KSTAT and projected into the reference space.

(B) Sorted expected expression reveals an s-shaped curve, where the elbow point distinguishes bins with negligible expression from those containing significant signal. Two linear models are fit to the data, and their intersection point is used to determine the threshold value for filtering.

(C) The filtered image highlights the location of the most significant Nphs1 expression.

Development • Supplementary information

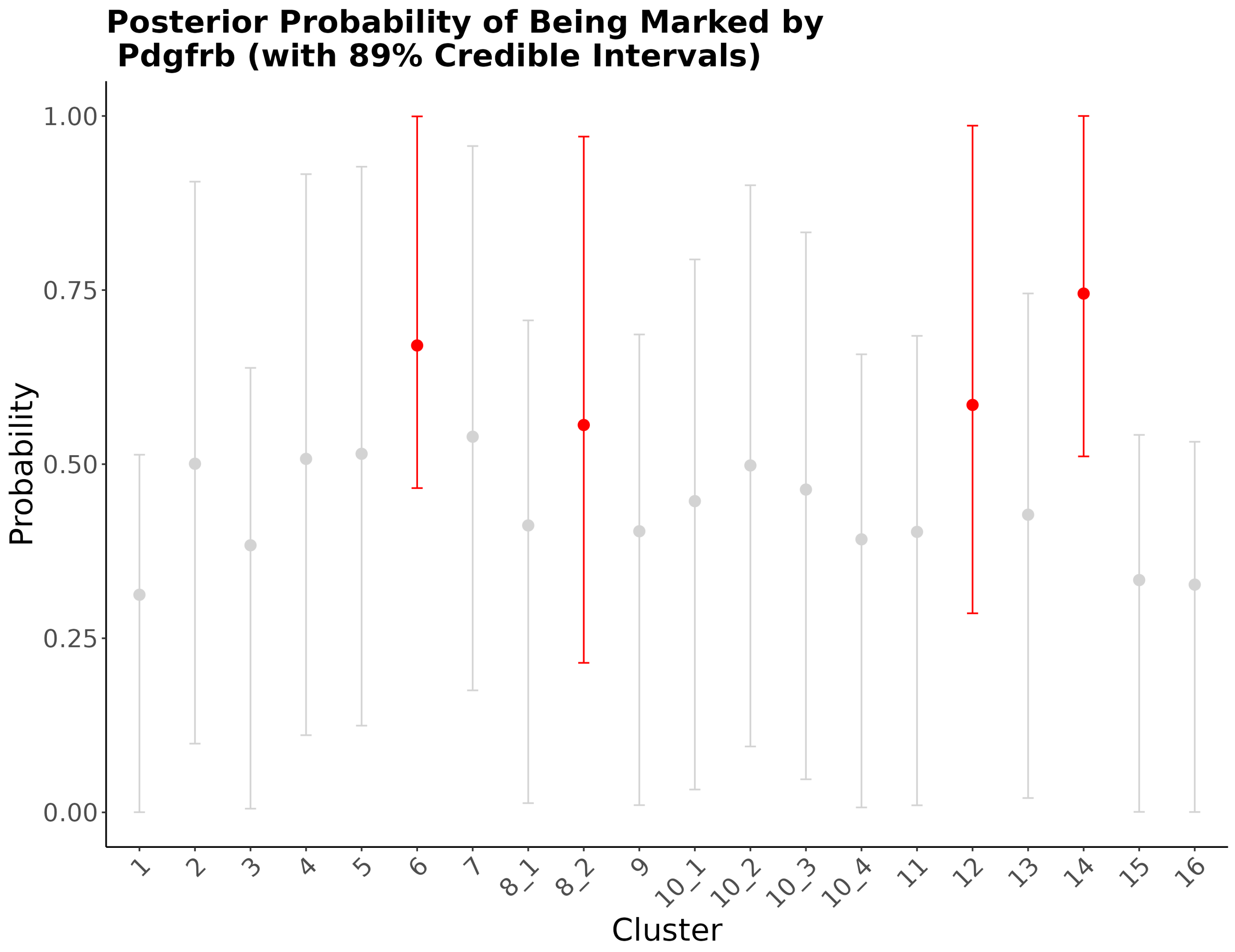

Fig. S11. Probability of cluster being marked by Pdgfrb expression.

We computed the posterior probability that each cluster of interstitial cells would be marked by Pdgfrb expression. This plot depicts the 89% credible intervals for this probability for each cluster. The intervals for the four clusters with the highest posterior probability are colored red.

Table S1. Reconstruction error for landmark genes.

Landmark gene	Reconstruction error (full)	p-value	Reconstruction error (LOOCV)	p-value
Ace	592.7803	0	794.9769	0
Acta2	840.4386	0	1281.791	0
Adamts2	1939.5471	0	4896.1777	0
Adamts5	231.19473	0	315.5603	0
Agtr1a	419.86465	0	1015.5008	0
Aldob	596.4809	0	869.4812	0
Apoe	4.276144	0	334.21423	0
Aqp1	2915.077	0	3034.2988	0
Aqp3	29.063541	0	1629.1128	0
Atp1b1	63.55609	0	245.17203	0
Bmp3	65.98732	0	985.7909	0
Bmper	121.84595	0	2735.4553	0.0004
C1qb	1173.8735	0	1385.0999	0
Calb1	89.899475	0	3965.7373	0
Ccnd1	42.59207	0	282.76263	0
Cdkn1c	409.6261	0	4034.3135	0
Cited1	1321.2715	0	1326.2123	0
Clca3a1	267.19098	0	894.39014	0
Cldn1	873.1045	0	574.28894	0
Cldn19	424.38123	0	624.3763	0
Col14a1	346.964	0	256.73468	0
Col4a4	1389.5942	0	2569.4248	0
Cpn1	1400.9858	0	1422.1536	0
Crabp2	7615.647	0.9469	1902.7458	0
Crlf1	3781.2483	0.0023	4123.222	0.0105
Cyp2j5	115.4169	0	638.25903	0
Dcn	2053.769	0	4953.993	0
Dkk1	487.45096	0	6825.018	0.0002
Dkk2	7547.0713	1	2904.4326	0
Edn1	2158.4153	0	847.7621	0
Egfl7	4.412018	0	377.74078	0
Egfl8	2017.7986	0	1977.4136	0
Epcam	285.5703	0	216.75189	0
Fabp4	141.49905	0	143.84642	0
Fibin	2416.9724	0	4172.3022	0.0012
Foxd1	224.92447	0	4997.4453	0.8156
Fxyd2	130.90854	0	276.71716	0
Fxyd3	363.2724	0	2576.2085	0
Gata3	39.589386	0	582.03204	0
Gm13889	896.47253	0	727.1924	0
H19	66.72705	0	383.74414	0
Irx1	73.47623	0	1409.5747	0
Itga1	1617.7755	0	1346.5057	0
Khdrbs3	523.4767	0	334.07495	0
Krt18	121.275345	0	215.85541	0
Krt19	512.81366	0	2845.2456	0
Lcn2	2290.026	0	1460.5105	0
Ldhb	312.76855	0	751.4701	0
Lox	11.50905	0	119.16601	0
Ly6a	527.48303	0	671.5596	0
Mafb	802.4355	0	974.58575	0
Mal	857.7024	0	1184.7786	0
Mgp	913.16	0	889.43634	0
Myh11	714.8053	0	15812.24	0
Ndufa4l2	5871.611	0	3011.6877	0
Nphs1	451.6538	0	771.3397	0
Npnt	550.5022	0	829.5585	0
Nrp2	688.3566	0	729.10803	0
Nts	246.242	0	576.1212	0
Pdzk1	606.09454	0	622.7868	0
Penk	39.309387	0	578.85425	0
Podxl	1.1750536	0	415.846	0
Prrx1	2674.9304	0	1285.189	0
Pttg1	3289.2952	0	2607.7266	0
Rbp4	656.0679	0	756.3583	0
Rgs5	1050.9075	0	2800.9683	0
Rprm	2956.7502	0.0005	10170.801	1
S100a8	5917.1797	0.0008	7430.7344	0.0205
S100g	2439.9175	0	3053.4954	0
Six2	412.72992	0	6960.844	0.1189
Slc27a2	3.3423166	0	539.2528	0
Slfn9	1587.6963	0	881.9777	0
Smoc2	420.83698	0	1763.409	0
Sox17	426.28735	0	761.8478	0
Stmn2	318.62225	0	6015.0625	0.0436
Sytl2	875.6852	0	765.98645	0
Tagln	308.05737	0	1722.0133	0
Tfdp1	234.39389	0	283.59528	0
Thbs2	63.78773	0	630.5244	0
Timm8a1	2602.7458	0	992.02814	0
Tm4sf1	774.6371	0	1640.4381	0
Twist1	1269.4514	0	3564.3967	0.0002
Tyms	90.73059	0	195.61588	0
Tyrobp	8824.809	0.0067	20546.783	0.3577
Wfdc2	1211.4172	0	1279.9431	0
Wt1	7788.66	0.0002	7883.96	0.0007

Development • Supplementary information

Table S2. Ligand-receptor interaction scoring.

Interaction ID	Interactors	Mean	Score	p-value	Interaction ID	Interactors	Ligand mass (l)	Receptor mass (m)	Sinkhorn divergence (d)	Likelihood (L)
CPI-SS0D0235DCE	Slit2-Robo2	14.696	100	0	CPI-SS068ACD209	Efna5-Ephb2	39832.30469	2954.366699	39.0512886	277.7886658
CPI-SS07660BCA2	Ntn1-Unc5c	4.914	100	0	CPI-CS0406B0B69	Lipa-Rora	15783.68359	8826.664063	47.89361191	246.4477844
CPI-SS075F456AF	Tenm3-Adgrl2	2.895	100	0	CPI-SC0B1EBCCC6	Bmp7-Bmpr1a+Bmpr2	25725.01367	4377.529785	43.65183258	243.1027679
CPI-SS04FD52559	Tenm3-Adgrl1	2.066	100	0	CPI-SS0579FA162	Igf2-Igf1r	428376.625	7988.079102	249.1118774	234.8224487
CPI-SS0FD44484F	Tenm4-Adgrl2	1.829	100	0	CPI-CS06C6527F4	Dhcr7-Rora	12272.48633	8826.664063	46.94306946	221.713974
CPI-SS0579FA162	Igf2-Igf1r	1.511	100	0	CPI-SS0703C3CE4	Dkk1-Lrp6	11209.10156	6030.263672	38.86930466	211.5177307
CPI-SS021718DBF	Tenm2-Adgrl2	1.303	100	0	CPI-SC0DA028FF9	Bmp7-Bmpr1b+Bmpr2	25725.01367	3126.866699	43.66055298	205.4202423
CPI-SS0EC12EAD9	Tenm4-Adgrl1	1	100	0	CPI-SC05824EDF4	Bmp7-Acvr2b+Bmpr1b	25725.01367	3126.78125	43.66101074	205.4152679
CPI-SS0F67B526A	Sema3c-Nrp2	0.713	100	0	CPI-SS04A97448D	Fgf1-Fgfr2	12015.25684	6004.87207	44.57022476	190.5783539
CPI-SS0C977FC22	Sostdc1-Lrp6	0.624	100	0	CPI-SS0CE693E3E	Lgals9-P4hb	19570.21094	3649.864258	44.49317551	189.9514771
CPI-SS008E98D3E	Flrt2-Adgrl1	0.502	100	0	CPI-SS043E22BF5	Vegfa-Nrp1	30518.94336	2584.794922	47.05712891	188.7436523
CPI-SS09159B142	Tenm2-Adgrl1	0.474	100	0	CPI-SS0E41702C1	Igf1-Igf1r	7935.09082	7988.079102	43.52529144	182.9175873
CPI-SS0ECC9DF3E	Efnb2-Ephb2	0.411	100	0	CPI-SC033AB13AE	Igf1-Gpc3+Igf1r	7935.09082	7987.899414	43.52529526	182.9154968
CPI-SS0593F278B	Agrn-Ptprs	0.367	100	0.176	CPI-SS075F456AF	Tenm3-Adgrl2	368941.25	4949.40332	244.8256226	174.541275
CPI-SC063989793	Wnt4-Fzd3+Lrp6	0.366	100	0	CPI-SS0FC01B947	Efna5-Epha4	39832.30469	1150.328735	39.1037178	173.1054077
CPI-SS046F8AD8B	Vegfa-Nrp2	0.347	100	0.32	CPI-SS028688B7B	Efna5-Epha7	39832.30469	1124.599365	38.9058876	172.0288544
**CPI-SC0B739B6BE**	**Wnt5a-Fzd3+Lrp6**	**0.304**	**100**	**0**	CPI-SS04FD52559	Tenm3-Adgrl1	368941.25	4456.830566	243.4148865	166.5883026
CPI-SS06E797E50	Efnb1-Ephb2	0.3	100	0	CPI-SS0755A26DD	Fgf1-Fgfr1	12015.25684	4585.426758	44.56425476	166.5596313
CPI-SS02A53F29E	Lrpap1-Sort1	0.261	100	0	CPI-SC069FF84F5	Spp1-Itga9+Itgb1	29533.34375	2189.715088	48.42755508	166.0571594
CPI-SC0D05B755E	Bmp6-Acvr2b+Bmpr1a	0.234	100	0	CPI-SS0D0235DCE	Slit2-Robo2	100192.0313	12865.54102	223.9519348	160.3155975
CPI-SC0A978EC39	Bmp6-Acvr1+Acvr2b	0.234	100	0	CPI-SS042FCC9BE	Nectin3-Pvr	28451.9082	1532.533936	42.03207397	157.1013641
CPI-SC0245AE3FF	Wnt4-Fzd2+Lrp6	0.226	100	0	CPI-SS042A6F835	Igf2-Igf2r	428376.625	3530.928711	249.0667725	156.1498413
CPI-SC0EB633CED	Wnt4-Fzd2+Lrp5	0.2	100	0.304	CPI-SS0EA023E51	Tenm3-Adgrl3	368941.25	3720.866211	244.6875458	151.4219208
CPI-SC0E11F543C	Wnt4-Fzd3+Lrp5	0.2	100	1	CPI-SS083BB1EB3	Tenm1-Adgrl2	7670.475586	4949.40332	42.07691193	146.4346008
CPI-SC04AF69F59	Wnt4-Fzd4+Lrp6	0.183	100	0.856	CPI-SS05A94DE17	Fgf2-Fgfr2	5302.749512	6004.87207	40.37108994	139.7757874
CPI-SC07D1540F8	Wnt5a-Fzd2+Lrp6	0.164	100	0	CPI-SS097A84265	App-Tnfrsf21	860734.4375	1408.94519	249.7185669	139.4539337
CPI-SS0AE964947	Gas6-Axl	0.147	100	0.16	CPI-SS085E3E37F	Tenm1-Adgrl1	7670.475586	4456.830566	41.96911621	139.3138733
CPI-SC0675C8528	Wnt5a-Fzd2+Lrp5	0.139	100	0.432	CPI-SC09B2D1C63	Tnc-Itga8+Itgb1	217285.9063	5154.230957	247.5878143	135.1663055
CPI-SC081B09E04	Wnt5a-Fzd3+Lrp5	0.139	100	1	CPI-SS046F8AD8B	Vegfa-Nrp2	30518.94336	1299.550293	47.09488678	133.7234344
CPI-SC0E149DD74	Wnt5a-Fzd4+Lrp6	0.121	100	0.92	CPI-SS07ACDDCD6	App-Sorl1	860734.4375	1300.815186	251.0952148	133.2612762
CPI-CS0406B0B69	Lipa-Rora	2.893	0	1	CPI-SS05632AB58	Ncam1-Fgfr1	181421.4375	4585.426758	220.1001892	131.042984
CPI-CS0D81115C8	Dhcr24-Rora	2.891	0	1	CPI-SS04E33D0FB	Efna5-Epha1	39832.30469	660.123291	39.18597794	130.8577728
CPI-CS06C6527F4	Dhcr7-Rora	2.866	0	1	CPI-SS063D79C85	Dkk2-Lrp6	5348.495117	6030.263672	43.76845169	129.7546082
CPI-CS06A0713BD	Cel-Rora	2.848	0	1	CPI-SS011931B0F	Sema4g-Plxnb2	12889.01563	3405.343018	51.2357254	129.3056335
CPI-SS0EA023E51	Tenm3-Adgrl3	2.557	0	1	CPI-SS0ECCB4382	Efnb3-Ephb2	11705.27148	2954.366699	46.12720108	127.4869766
CPI-SS0A932EB77	Slit2-Robo1	2.385	0	0.528	CPI-SS0D5E30C6C	Nrg3-Erbb4	10710.28516	2275.010254	38.81362152	127.1768417
CPI-SS081E1A895	Lrfn5-Ptprd	2.374	0	1	CPI-SS038A612D7	Tenm1-Adgrl3	7670.475586	3720.866211	42.11869812	126.8405838
CPI-SS0FA9304CE	Lrfn4-Ptprd	2.354	0	1	CPI-SC017215F7F	Gdf11-Acvr2b+Tgfbr1	10766.84082	2759.532227	44.34699249	122.9129639
CPI-SS0E0D00210	Lrrc4b-Ptprd	2.344	0	1	CPI-CS036B95798	Ptges3-Ptger4	498555.6875	1901.497803	251.0640106	122.6366501
CPI-SS08069FE13	Nlgn3-Ptprd	2.344	0	1	CPI-SS01F67987A	Fgf2-Fgfr1	5302.749512	4585.426758	40.40019226	122.055397
CPI-SS0522C3A29	Slitrk1-Ptprd	2.341	0	1	CPI-SS00508D1C2	Vegfb-Nrp1	15260.83691	2584.794922	52.10517883	120.5372696
CPI-SS09B85D556	Slitrk2-Ptprd	2.339	0	1	CPI-SC050D66C45	**Wnt9b-Fzd3+Lrp6**	**7171.442871**	**3704.033203**	**42.88238525**	**120.1881866**
CPI-SS096F8C268	Ptprd-Il1rap	1.99	0	1	CPI-CS05B3376EE	B2m-Il6st	17259.17578	1537.008545	44.04733276	116.9307022
CPI-SS0F9F83827	Ptprd-Il1rapl1	1.989	0	1	CPI-SC024FAED24	Lamc1-Itga6+Itgb1	621825.5625	1319.977783	247.0303497	115.9757767
CPI-SS05632AB58	Ncam1-Fgfr1	1.942	0	0	CPI-SS039863E62	Ccl25-Ccr9	472653.0625	1763.671265	250.3094025	115.3460922
CPI-SS01F00A48A	Tenm4-Adgrl3	1.491	0	1	CPI-CS086D3E39F	Dhcr7-Nr1h2	12272.48633	2321.571045	46.99690247	113.5763855
CPI-SS0C21302BC	Ncam1-Agrn	1.481	0	1	CPI-SS0C70ADA28	Efna5-Epha3	39832.30469	492.5586243	39.12938309	113.1992874
CPI-SC033AB13AE	Igf1-Gpc3+Igf1r	1.415	0	1	CPI-CS040CBA095	Gls+Slc17a6-Grm7	560953.625	1467.125977	253.818222	113.0249634
CPI-SS0E41702C1	Igf1-Igf1r	1.415	0	1	CPI-CS029D39F39	Gls+Slc17a6-Gria3	560953.625	1450.583984	252.6257019	112.9164963
CPI-SS068ACD209	Efna5-Ephb2	1.115	0	0	CPI-SC045BF427E	Wnt9b-Fzd2+Lrp6	7171.442871	3054.67334	43.01111603	108.8190536

Development • Supplementary information
